# Functional annotation by identification of local surface similarities: a novel tool for structural genomics

**DOI:** 10.1186/1471-2105-6-194

**Published:** 2005-08-02

**Authors:** Fabrizio Ferrè, Gabriele Ausiello, Andreas Zanzoni, Manuela Helmer-Citterich

**Affiliations:** 1 Boston College, Biology Department, Chestnut Hill MA, USA; 2 Centre for Molecular Bioinformatics, Department of Biology, University of Rome Tor Vergata, Italy

## Abstract

**Background:**

Protein function is often dependent on subsets of solvent-exposed residues that may exist in a similar three-dimensional configuration in non homologous proteins thus having different order and/or spacing in the sequence. Hence, functional annotation by means of sequence or fold similarity is not adequate for such cases.

**Results:**

We describe a method for the function-related annotation of protein structures by means of the detection of local structural similarity with a library of annotated functional sites. An automatic procedure was used to annotate the function of local surface regions. Next, we employed a sequence-independent algorithm to compare exhaustively these functional patches with a larger collection of protein surface cavities. After tuning and validating the algorithm on a dataset of well annotated structures, we applied it to a list of protein structures that are classified as being of unknown function in the Protein Data Bank. By this strategy, we were able to provide functional clues to proteins that do not show any significant sequence or global structural similarity with proteins in the current databases.

**Conclusion:**

This method is able to spot structural similarities associated to function-related similarities, independently on sequence or fold resemblance, therefore is a valuable tool for the functional analysis of uncharacterized proteins. Results are available at

## Background

Detection of sequence or fold similarity is often used to infer the function of uncharacterized proteins. By this approach one can tentatively assign a function to approximately 45–80% of the proteins identified by the genomic projects [1,2]. However, function is mostly determined by the physical, chemical and geometric properties of the protein surfaces [3,4], and cases have been described where the same local spatial distribution of residues important for function is achieved with apparently unrelated structures and/or sequences [5]. One of the best known examples is represented by the SHD catalytic triad of serine proteinases [6-8]. Furthermore, surface similarities have been detected in unrelated ATP/GTP binding proteins [9,10] and in the guanine binding sites of p21Ras family GTPases or in the RNA binding site of bacterial ribonucleases [10]. By local structural comparison Hwang *et al*. [11] were able to infer correctly the nucleotide binding ability of an uncharacterized *Methanococcus jannaschii *protein.

On the other hand, similar folds can have different functions if their active sites have diverged [12-15]. As a consequence, methods purely relying on sequence and global structure comparison may lead to inaccurate function-related annotations in cases in which few residues are responsible for the specificity of substrate interaction.

The vast majority of well-studied functions (enzymatic activities, binding abilities etc.) are encoded by a relatively small set of residues, often not contiguous in the protein sequence but organized in a conserved geometry on the protein surface that may be used as a marker for reliable functional annotation. Although exposed to the solvent, these function-related residues are often located in surface clefts or cavities [16]. Such residues define functional modules conserved in some proteins sharing a molecular function even if differing in sequence and structure. Several tools for discovering conserved three-dimensional patterns in protein structures have already been proposed [17-20]. Schmitt *et al*. [21] developed a clique-based method to detect functional relationships among proteins. This approach does not rely on detection of sequence or fold homology and highlights a number of non-obvious similarities among protein cavities. The algorithm, however, is computationally intensive and cannot be applied to an all-against-all analysis of protein surface regions. Binkowski and co-workers [22] recently described an approach for detecting sequence and spatial patterns of protein surfaces: the underlying algorithm is fast, but cannot identify similarities that are independent of the residue order in the compared proteins. Two related papers [23,24] describe a method for local structural similarity detection, which is of great relevance since it is able to evaluate the statistical significance of each match. This method (PINTS) has been then used to analyze protein structures from structural genomics projects [25]. Other recent papers present algorithms able to find structural motifs possibly related to a function and to use them to scan protein structure libraries [26-31].

In a previous work [32] we described the construction of a non redundant library of surface annotated functional sites and a fast comparison algorithm able to find structural similarities independently on the residue sequence order. We report here the analysis of the results of the first all-versus-all comparison of the protein functional sites, the validation of the comparison procedure in a test dataset and its application for annotating a dataset composed of proteins solved in structural genomics projects. The results are available for experimental test at the address .

## Results and discussion

### Functional sites comparison

**Figure 1:**
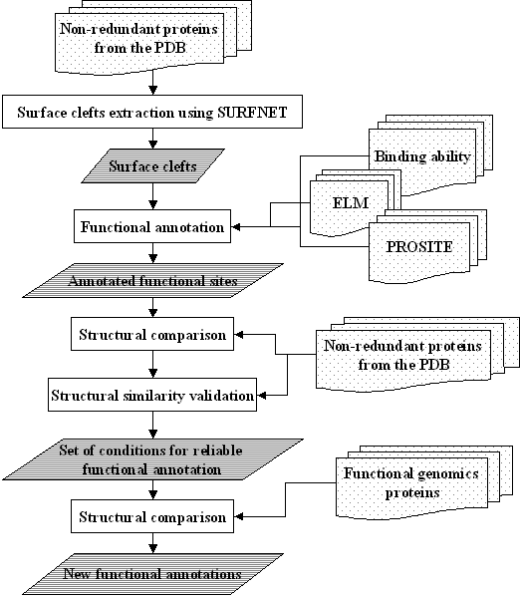
**Description of the experimental procedure. **Surface functional sites are automatically located and annotated as described in Methods. Surface clefts, identified by means of SURFNET, are filtered using a volume threshold, and annotated for the binding ability or for the presence of a functional motif from the PROSITE or ELM databases. This library (the SURFACE database) is used to scan a non-redundant collection of protein structures; a semi-automated procedure is used to define conditions for which the structural similarity implies also a functional relationship. Finally, the SURFACE database is used to analyze a list of proteins with unknown function from structural genomic projects, obtaining in several cases significant similarities that could have not been spotted through sequence or fold similarity.

We used the *compendium *of protein surface regions associated to molecular functional sites stored in the SURFACE database [32]. This is a collection of 1521 annotated functional regions obtained following the procedure described in Figure 1 and in the Methods section. Each patch has at least a function-related annotation, that may be the ability to bind a certain ligand, or a match with a PROSITE or ELM pattern [33,34]. Ligand-binding abilities are included among gene ontology (GO) molecular functions [35], as well as many PROSITE patterns and ELM motifs. Some other PROSITE patterns correspond to short motifs that are conserved in all members of certain protein families, which not necessarily are associated to known function-related residues. We chose to include this class of patterns in our annotation system, since they offer a quick way to verify the reliability of a match, and in many cases these motifs do contain functional residues. Hence, our annotations can be classified either as molecular functions or protein signatures. It is worth noticing that the annotation is extended to the whole patch but is also assigned to a subset of specific annotated functional residues.

In [32] the structural matches obtained from the comparison of he SURFACE library against the entire collection of surface clefts (both annotated and not annotated) were evaluated by means of the Z-score of each match length against the distribution of the match lengths for any given annotated patch. Here we perform an exhaustive analysis in order to find conditions for which a structural similarity also suggests a function-related similarity. First, only those matches which include annotated functional residues are considered, therefore each structural similarity match is likely to hold a functional meaning. This step is crucial since many matches may be obtained because of general fold similarity, without an underlying functional relationship. Finding a *functional *match induces an annotation of at least some of the residues, and suggests reasonable hypotheses as to function (we are currently investigating how to use our approach to find *novel *function-related structural motifs, i.e. recurrent structural matches between proteins that can not be explained only by fold similarity and that may imply a previously undetected functional similarity).

From the comparison of the SURFACE library against the entire collection of surface clefts, we collected a grand total of 65910 stringent matches among patch pairs, about 4.5% of which involve 6 or more residues and 4.5% involve 10 or more residues. A not negligible amount of these matches involve residue pairs whose relative distance is not conserved in the corresponding protein sequences. More interestingly, some of the matches involve residues whose sequence order and/or sequence spacing is different in the two proteins: some of these cases, that may be examples of convergent evolution, are currently under investigation. As an example, metals can interact with proteins by means of similar arrangements of residues that can be found across different folds [36-38]. Scanning our dataset with zinc-binding patches leads to the finding of significant matches to proteins belonging to 42 different folds and 6 different classes as defined by SCOP [39]. Different metal-binding patches lead to similar findings, even though less dramatic. Further analysis would suggest how many of these cases are associated with functional similarities as well.

The fraction of matches validated (as described in the Methods section) sensibly increases with the Z-score (Table 1). At lower Z-scores, the GO terms and SWISS-PROT keywords validation methods are more represented, while, for more significant matches, ability to bind the same ligands, fold similarity and co-presence of PROSITE motifs become more relevant.

**Figure 2:**
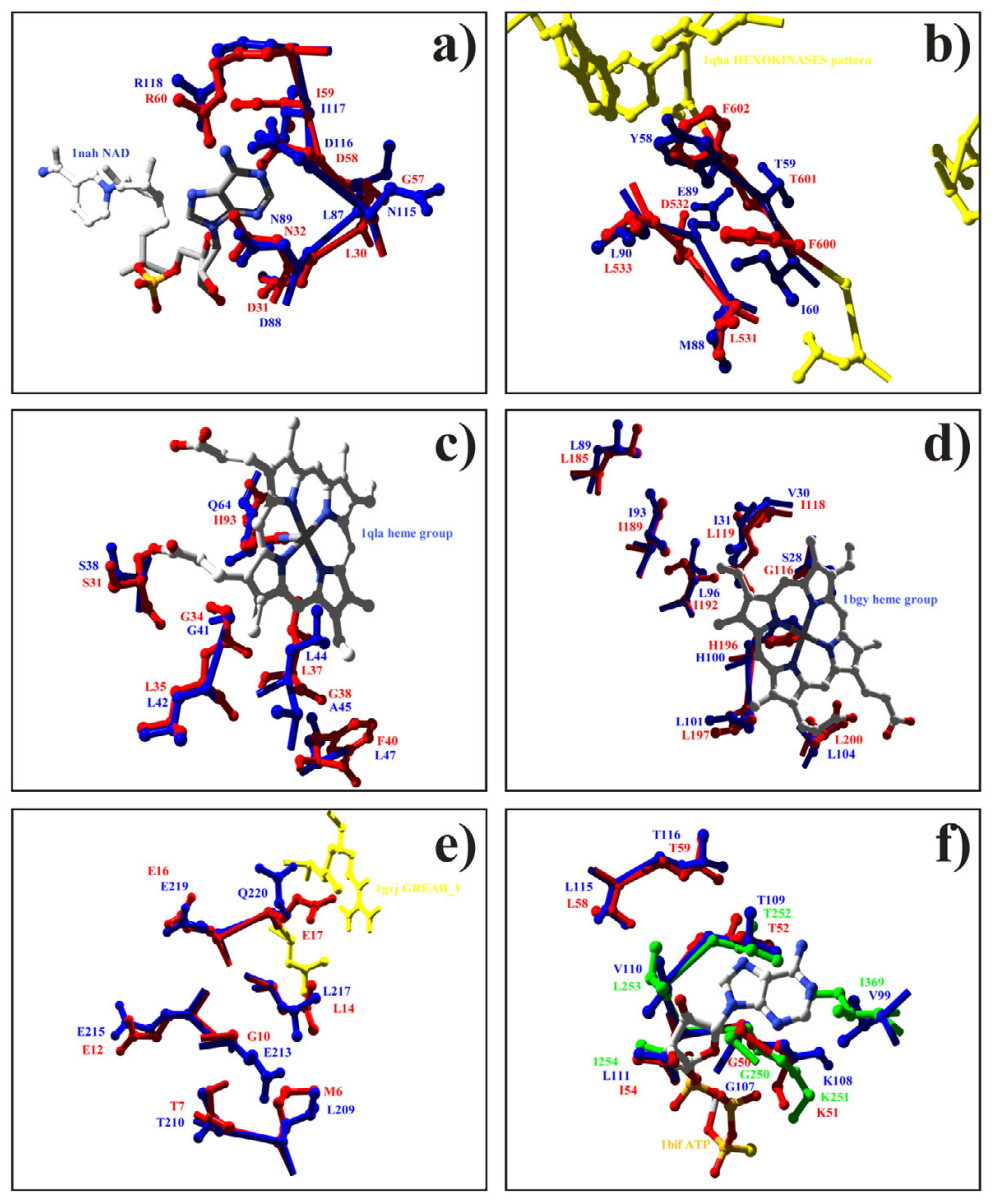
**Significantly matching residues on proteins sharing no structural or sequence similarity. **Similarity detected comparing the SURFACE database of annotated functional sites against a list of annotated monomers (a,b) or proteins with unknown function from structural genomics projects (c,d,e,f); the annotated patch residues are colored in blue, the matching residues in red; whenever possible, the patch annotation (bound ligand or PROSITE pattern) is shown. (a) Similarity detected between the *E. coli *UDP-galactose 4-epimerase (PDB code 1nah) NADH-binding patch and the *H. influenzae *YecO methyltransferase (1im8); the NAD co-crystallized with 1nah is shown; the similarity involves 7 residues (with a Z-score 9.06). (b) Structural similarity between the HEXOKINASES PROSITE pattern-annotated patch of the human hexokinase type I (1qha) and the bacteriophage ms2 capsid protein; additional 1qha annotated residues are shown in yellow. (c) Structural similarity detected between the *B. subtilis *Yqvk protein, and the *Wolinella succinogenes *fumarate reductase cytochrome B subunit heme group binding patch. (d) Match between Hi1480 protein from *Haemophilus influenzae *and the bovine cytochrome Bc1 heme-binding patch. (e) Similarity between the *B. subtilis *protein Yqeu and the *E. coli *Grea transcript cleavage factor GREAB_1-annotated patch; additional pattern-annotated residues are shown in yellow. (e) Similarity between *E. coli *lysozyme inhibitor and two ATP-binding patches, the *Rattus norvegicus *6-Phosphofructo-2-Kinase/ Fructose-2,6-Bisphosphatase major patch (red) and the mouse Aaa ATPase P97 (green).

The matches that cannot be structurally or functionally justified by these methods and that are characterized by a high Z-score are relatively few (see Table 1). 171 matches out of 2173 (7.9%) having a Z-score higher than 7 are not validated following the above mentioned criteria (Table 1). Of these 171 matches, 130 can be considered as true positive matches, confirmed by literature and information derived from different sources and databases. The remaining 41 matches (1.9%) are not confirmed and should be tested experimentally. About 2% of the highly significant matches can be considered as possible false positive hits or new annotations. Some of these cases are shown and discussed in Figure 2(a,b).

**Table 1:** Structural matches Z-score distribution and validation. This Table shows the number of structural matches (second column from the left) found as a function of the Z-score of the match. The third column from the left (labeled "validated") reports the number of matches for which at least one of the validation criteria holds. The following columns show a breakdown of the number of matches validated by each validation condition (from the fourth column on the left to the rightmost: same PROSITE pattern annotation; same binding ability; common GO term annotation; same SCOP fold; same Enzyme Classification number; sequence similarity at least 40%; common SwissProt keyword). Note that the sum of the matches validated by the different criteria for each row is higher than the total number of validated matches at that given Z-score, since some matches can satisfy more than one condition. At increasing Z-scores, the ratio of validation condition that we consider less reliable (SwissProt keywords, GO terms) decreases, while the ratio of more reliable annotations (i.e. same binding ability, same PROSITE pattern annotation) increases.

Z-score	Total	Validated	PROSITE	Ligand	GO	Scop	E.C.	Seq. sim.	SwissProt kw
3.0	31341	7066	366	951	3565	765	99	2	5655
3.5	14948	4002	747	830	2222	889	48	3	2944
4.0	9721	2814	557	613	1680	788	44	1	2043
4.5	3942	1346	440	467	841	390	32	1	989
5.0	1549	764	281	234	436	411	5	1	514
5.5	976	612	287	181	320	399	7	0	342
6.0	639	457	177	209	267	271	3	0	323
6.5	621	548	279	258	298	447	4	0	383
7.0	365	328	157	115	180	246	2	0	200
7.5	260	219	105	68	109	176	6	1	152
8.0	270	238	104	87	149	191	0	1	169
8.5	209	195	80	57	129	153	8	1	131
9.0	122	107	54	54	70	87	0	0	63
9.5	137	129	60	48	74	119	0	0	80
10.0	124	113	53	61	75	104	0	1	86
10.5	55	51	17	22	29	43	2	0	36
11.0	106	103	46	40	65	91	4	0	66
11.5	88	88	42	43	65	80	5	0	55
12.0	78	77	33	34	51	75	5	0	52
12.5	71	69	26	32	38	64	5	1	54
13.0	49	47	30	21	24	45	0	0	30
13.5	39	39	9	19	17	39	1	0	24
14.0	29	29	14	16	18	29	3	0	25

From this validation procedure the emerging result is that, using stringent parameters in the comparison step and using the Z-score as a threshold, our algorithm is reliable and able to spot local structural similarities related to functional relationships with only few non confirmed hits, which can be considered as false positives or as testable hypotheses.

An estimation of *false negative *matches (defining false negative match as the missing detection of structural similarity between two proteins sharing the same function) is not immediate, for the reason that the same or similar molecular function may be achieved in different ways using a different three-dimensional residue arrangement. We estimated the occurrence of false negatives for PROSITE annotated patches, using the list of known true positives (for which the function encoded by the pattern is experimentally verified) for each pattern that is provided by PROSITE. The procedure is done as follows: for all the patches annotated with a given PROSITE pattern, we collect all matches obtained scanning with these patches the entire patches dataset, selecting only those matches having Z-score higher than a fixed threshold. The fraction of known true positives that are not found using the pattern-annotated patches as queries (i.e. the false negatives), when retrieving only those matches having Z-score higher than 5, is 0.3 (meaning that we are able to correctly retrieve the 70% of the occurrences of PROSITE patterns in the dataset), and it raises to 0.35 setting the Z-score threshold to 7.

### Benchmark cases

**Figure 3:**
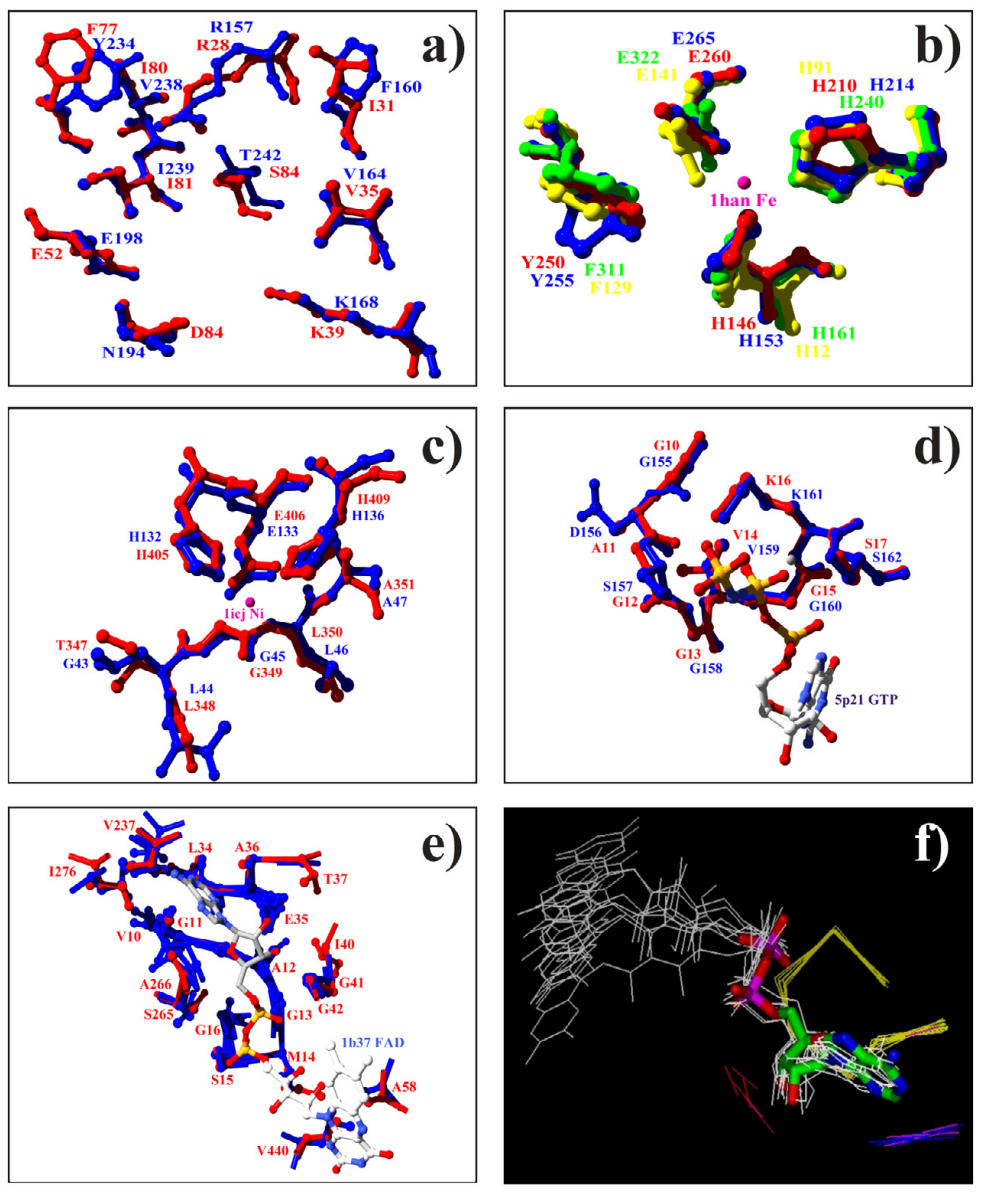
**Benchmark cases analysis. **(a) Structural superposition of the *S. cerevisiae *(red) and the *E. coli *(blue) chorismate mutase (PDB code 4csm and 1ecm, respectively). These two patches align ten residues, with a resulting Z-score of 15.76. (b) Structural superposition of the 4-hydroxyphenylpyruvate dioxygenase (PDB code 1cjx, red), the 2,3-dihydroxybiphenyl 1,2-dioxygenase (1han, blue), catechol 2,3-dioxygenase (1mpy, green) and the methylmalonyl-Coa epimerase (1jc5, yellow). The 1han co-crystallized iron ion is shown. (c) Superposition of the tumor necrosis factor-alpha-converting enzyme (1bkc, red) and the peptide deformylase (1icj, blue). The 1icj co-crystallized nickel ion is shown. (d) Structural superposition of the human P21 ras protein (5p21, red) and HprK/P 1jb1 (blue). (e) Structural superposition of the 1b37 FAD-binding pocket (red) with the highest-score matches obtained in a database search (blue). The 1b37-bound FAD is shown. (f) Bound ligands superposition. Using the three-dimensional transformation used to superpose the residues aligned in (e), also ligands that are bound to some of these proteins are consequently superposed. The ADP molecule bound to the 1djn patch nicely matches the ADP moiety in the similar FAD-binding pockets.

To further test the ability of the procedure in finding known cases of functional similarities among proteins for which sequence and/or structure similarity is not significant, a number of benchmark cases were investigated (Figure 3):

i) The *S. cerevisiae *and the *E. coli *chorismate mutase (PDB codes: 1ecm and 4csm, respectively), despite the very low sequence identity, share a similar fold and a similar main functional site [18,21]. The 1ecm largest patch is annotated for the oxy-bridged prephenic acid binding ability. Using this patch as a query, the highest Z-score match is found with the 4csm largest patch (Figure 3a).

ii) The Glyoxalase/Bleomycin resistance protein/Dihydroxybiphenyl dioxygenase fold is common to several unrelated metal ion binding proteins sharing similar catalytic mechanisms, including the bleomycin resistance protein, glyoxalase I, and a family of extradiol dioxygenases [40]. We detected a significant similarity among *P. fluorescens *4-hydroxyphenylpyruvate dioxygenase (PDB code 1cjx), *B. cepacia *2,3-dihydroxybiphenyl 1,2-dioxygenase (1han), *P. putida *catechol 2,3-dioxygenase (1mpy) and *P. shermanii *methylmalonyl-Coa epimerase (1jc5). The comparison algorithm correctly identifies the residues involved in Fe binding (Figure 3b). 1han second largest patch is annotated for the iron binding ability. Structural matches with 1mpy, 1cjx and 1jc5 functional sites are found at high Z-score (7.19).

iii) Metal ions can be coordinated by histidine clusters. We identified a similarity between the human tumor necrosis factor-alpha-converting enzyme (PDB code: 1bkc) Zn binding site and the *E. coli *peptide deformylase (PDB code: 1icj) Ni binding site, despite their sequence and fold diversity (Figure 3c). The zinc-binding patch of 1bkc shares eight residues in the same structural conformation with the nickel-binding patch of 1icj, with a Z-score of 10.66.

iv) Nucleotide binding abilities can be associated with several unrelated proteins; we detected a high-scoring match between the GTP-binding annotated patch of the human p21 ras protein (5p21) and the *L. casei *Hpr kinase (1jb1) that aligns eight residues with a Z-score of 9.01 (Figure 3d). These two proteins do not share any significant sequence or fold similarities.

As a further test, we analyzed the flavin-adenine dinucleotide (FAD) binding pockets, known to share structural similarities with other adenine-containing nucleotide binding pockets, despite sequence and fold differences [41,42]. FAD consists of an adenosine monophosphate (AMP) linked to a flavin mononucleotide (FMN) through a pyrophosphate bond and is involved as a cofactor in many biological processes. Using the FAD-binding patch of the *Zea mays *polyamine oxidase (1b37) as a bait, we selected 9 prey patches with Z-score higher than 12: 8 preys are annotated as being able to bind a FAD molecule and belongs to the same SCOP fold (FAD/NAD(P)-binding domain). The remaining *trapped *patch is the biggest patch of the trimethylamine dehydrogenase from *Methylophilus methylotrophus *(1djn), an iron-sulfur flavoprotein, and it is annotated as ADP-binding. 1djn is co-crystallized also with a FMN, which is very similar to FAD, but this ligand is associated to the second largest patch of the 1djn structure. The residues, which were associated by the alignment program, are shown in Figure 3e. These proteins share a very low sequence similarity, which cannot be revealed using BLAST2 [43]. The ADP binding patch of the 1djn structure is nicely superposed to the other patches in the binding pocket (Figure 3e), but shares no evident fold similarity with the other ones, and belongs to a different SCOP fold (the nucleotide-binding domain). When the selected structures in Figure 3f are physically superposed (finding the least-square fitting of the matching residues), also the ligands bound to these structures turn out to be nicely superposed. The procedure could therefore highlight the ability to bind a subset of the FAD molecule, namely an ADP molecule in the 1djn major patch, even with very low levels of sequence and structure similarity. Using each FAD binding patch to scan the dataset, we selected only proteins for which known functional properties are consistent with the FAD or nucleotide binding ability.

### Structural genomics proteins analysis

With the stringent parameters described above, we were able to detect only matches linked to function-related similarities, even in cases of non-homologous proteins. For that reason, once proved to be reliable, the procedure can be applied as a predictive tool to obtain clues concerning the function(s) of uncharacterized proteins.

We selected 257 protein structures from the PDB, corresponding to 513 chains that are marked as being of unknown function, or for being a hypothetical protein or for having been solved within a structural genomics project. We analyzed these structures by looking for reliable similarities to our functional sites library and were able to suggest one or more molecular functions to 191 of these chains, for a total of 534 similarity matches. For each match, we checked if the previously described criteria hold (i.e. common GO term, SwissProt keyword, EC number or SURFACE annotation). If not, a literature search has been done to verify the functional relationship. By means of this analysis of the likelihood of each single match, we found that 322 (the 60.3%) of these hits are validated by experimental analysis that have already characterized many of these proteins, while only 29 matches (5.4%) are not found confirmed in previous findings; 107 (20%) hits involve proteins for which the functions are still unknown; 76 hits (14.2%) involve proteins for which a hypothetical function has been assigned by means of sequence or structure global similarity. In this latter case, the function-related annotation obtained from our method can be considered as a new functional annotation that corrects or improves the actual function assignment. Hence, we were able to propose a function by similarity using the annotated patch database 184 times, to 127 different chains (matches with Z-score at least 7 are shown in Table 2). 56% of these new functional annotations are about a PROSITE pattern, the remaining 44% about a ligand binding ability; this is somewhat surprising, since the majority of the patches annotations in the SURFACE library regards binding abilities. A selection of the proposed functional regions is shown in Figure 2(c,d,e,f), while the complete list can be found at . For each match we tested the BLAST2 pair-wise sequence similarity between the sequence of the protein to which the query patch belongs and the target protein sequence, the PsiBLAST sequence similarity matches obtained by running the target sequence versus the non-redundant SwissProt+TrEMBL sequence database, the global structural similarities of the target structure in the PDB using SSM, and the local similarity using PINTS [24]. The match with the highest Z-score (14.29) is between the *B. subtilis *Yqvk protein (PDB code 1rty), and the *Wolinella succinogenes *fumarate reductase cytochrome B subunit major patch (1qlaC1), annotated with the heme group binding ability; the structural similarity involves 7 residues. The two proteins do not share any sequence or structural similarity, as checked using BLAST and the structural comparison algorithm SSM [44]. A PsiBLAST run of the Yqvk sequence against the non-redundant SwissProt+TrEMBL shows a significant similarity (E-value 4e-19) with the mouse cobalamin adenosyltransferase (SwissProt entry name MMAB_MOUSE), while the SSM comparison against the whole PDB leads to only one significant similarity, with another uncharacterized protein, the conserved protein 0546 From *Thermoplasma acidophilum *(1nog). A PINTS comparison [24] of Yqvk, against pre-compiled libraries of structural patterns, retrieves as most significant matches one with the human Small Nuclear Ribonucleoprotein Sm D3 (PDB code 1d3b), aligning 3 pairs of residues with r.m.s.d 0.32 and E-value 0.00481, and another with the pig Dihydropyrimidine Dehydrogenase 1htx (3 pairs aligned with r.m.s.d. 0.337 and E-value 0.00839). The heme binding ability thus may be a new functional annotation of this poorly known protein. The second highest Z-score match (13.32, 9 residues structurally aligned) occurs between Hi1480 protein from *Haemophilus influenzae *(1mw5) and the bovine cytochrome Bc1 heme-binding patch (1bgyC2). No significant sequence similarity is found in the SwissProt+TrEMBL (the highest match, whose E-value is 2.1, involves the putative *E. coli *RNA helicase, SwissProt entry name RHLE_ECOLI), as well as no significant matches are found using SSM. PINTS matches involving three residues are found with the virus influenzae Bha/Lsta protein (1mqm) and the *Candida tropicalis *Enoyl Thioester Reductase 2 (1h0k), whose E-values are 0.401 and 0.451, respectively. Another high-score match (Z-score 10.05, length 7 residues) is found between the *B. subtilis *protein Yqeu (1vhk) and the *E. coli *Grea transcript cleavage factor major patch (1grj_1), which is annotated with the GREAB_1 PROSITE pattern, a signature of this class of cleavage factors. Yqeu share SSM-detected structural similarities with another unknown-function protein (namely *H. influenzae *1nxz) and significant sequence similarity with a list of hypothetical and uncharacterized bacterial proteins. PINTS reports a local structural similarity with the zinc-binding site of the *E. coli *CTP-ligated T state aspartate transcarbamoylase (E-value 0.00894, r.m.s.d 0.544 over three pairs of residues).

**Table 2:** Non-validated functional annotations of non-annotated surface patches. Functional annotated sites have been compared to a collection of surface patches extracted from a non-redundant PDB subset. The reliability of each match was estimated via a series of criteria, as described in the text. The remaining similarities may be new functional annotations of uncharacterized functional sites, or false positive matches, and are shown in this table. Columns:*(i) *PDB code, chain name and patch number in the annotated query patch; *(ii) *Description of the protein to which the query patch belongs; *(iii) *Query patch functional annotation; *(iv) *Target patch; *(v) *Description of the protein to which the target patch belongs; *(vi) *Z-score of the match; *(vii) *SSM Q score; *(viii) *SSM P score; *(ix) *SSM Z score. The SSM Q score takes into account the number of aligned residues, their r.m.s.d. and the size of the proteins; a high Q score means a good similarity. The SSM P score is the log of the pValue (the probability that the match occurred by chance); P scores higher than 3 are considered significant by the authors of the method.

Patch 1	Protein	Patch 1 Annotation	Patch 2	Protein	Z-score	SSM Qscore	SSM P-value	SSM Z-score
3mdeA1	Acyl-CoA dehydrogenase	LIG_CO8	1g5bB6	Bacteriophage lambda S/T Protein Phosphatase	9.59	0.01	0	0.5
1qhaA2	Hexokinase I	HEXOKINASES	1i78A5	Outer Membrane Protease Ompt	9.44	0.01	0	0.5
1qhaA2	Hexokinase I	HEXOKINASES	1zdhA2	Bacteriophage Ms2 Protein Capsid	9.44	0.01	0	0.1
1bp1_1	Bactericidal permeability-increasing protein	LIG__PC	1qlwA2	Bacterial esterase 713	9.07	0.01	0	1.5
1nah_1	UDP-galactose 4-epimerase	LIG_NAD	1im8A1	YecO methyltransferase	9.06	0.1	0	4
4blcA1	Beef liver catalase	LIG_NDP	1io1A5	Phase 1 Flagellin	8.86	0.01	0	1.4
1dbtA1	Orotidine 5'-Monophosphate Decarboxylase	OMPDECASE	1dj8A1	E. Coli Periplasmic Protein Hdea	8.76	0.03	0	1.9
1fp2A1	Isoflavone O-Methyltransferase	LIG_SAH	1nah_1	UDP-galactose 4-epimerase	8.6	0.05	0	5.5
1fps_1	Prenyltransferase Trimethylamine	POLYPRENYL_SY NTHET_1	1h6gA2	Alpha-catenin Molybdopterin Biosynthesis Moeb	8.54	0.04	0	0.3
1djnA1	dehydrogenase	LIG_ADP	1jwbB1	Protein	8.51	0.05	0	5.3
19hcA1	Cytochrome C	LIG_HEM	1umuB1	UmuD' protein	8.44	0.03	0	4.2
1qhaA1	Type I Hexokinase	HEXOKINASES	1e2uA1	Hybrid Cluster Protein	8.34	0.01	0	0.1
256bA1	Cytochrome B562	LIG_HEM	1gpjA1	Glutamyl-tRNA reductase	8.25	0.05	0	0.4
1ep1B1	Dihydroorotate Dehydrogenase B	LIG_FAD	1pmi_8	Phosphomannose Isomerase	8.18	0.02	0	0.3
1tsdA1	Thymidylate synthase	LIG_U18	1prhA1	Prostaglandin H2 Synthase-1 Formylmethanofuran: Tetrahydromethanopterin	8.16	0.01	0	0.1
2nlrA1	Endoglucanase	LIG_G2F	1ftrA1	Formyltransferase	8.05	0.02	0	0.5
1ej0A1	RNA Methyltransferase	LIG_SAM	2cmd_1	Malate Dehydrogenase	8.01	0.12	0	3.9
1ecmB1	Chorismate mutase	LIG_TSA	1b3qB1	Histidine Kinase Chea	7.96	0.02	0	2.8
1av6A3	Vaccinia Methyltransferase Vp39	LIG_SAH	1b3mA1	Sarcosine oxidase	7.95	0.02	0	2.8
1av6A3	Vaccinia Methyltransferase Vp39	LIG_SAH	1b4vA1	Cholesterole oxidase	7.95	0.02	0	0.9
1qrrA1	Sulfolipid Biosynthesis (Sqd1) Protein	LIG_NAD	1g6q12	Arginine methyltransferase HMT1	7.85	0.04	0	1.9
1qrrA1	Sulfolipid Biosynthesis (Sqd1) Protein	LIG_NAD	1im8A1	YecO methyltransferase	7.85	0.09	0	2.4
1qrrA1	Sulfolipid Biosynthesis (Sqd1) Protein	LIG_NAD	1khhA1	Guanidinoacetate methyltransferase	7.85	0.1	0	2.9
6reqA1	Methylmalonyl-Coa Mutase	LIG_3CP	1fepA2	Ferric Enterobactin Receptor	7.79	0.01	0	0
6reqA1	Methylmalonyl-Coa Mutase	LIG_3CP	1jihB10	Yeast DNA Polymerase Eta	7.79	0.01	0	1
1bgyC1	Cytochrome BC1	LIG_HEM	1dc1B2	Bsobi Restriction Endonuclease	7.62	0.01	0	0.4
1bgyC2	Cytochrome BC1	LIG_HEM	1k92A4	Argininosuccinate Synthetase	7.62	0.01	0	0.2
1bgyC2	Cytochrome BC1	LIG_HEM	5r1rA2	Ribonucleotide Reductase R1	7.62	0.01	0	0.9
1qanA1	Rrna Methyltransferase Ermc'	RRNA_A_DIMETH	1b37B1	Flavin-dependent polyamine oxidase	7.54	0.04	0	5.3
1qanA1	Rrna Methyltransferase Ermc'	RRNA_A_DIMETH	1b3mA1	Sarcosine oxidase	7.54	0.04	0	4.3
1qanA1	Rrna Methyltransferase Ermc'	RRNA_A_DIMETH	1gpeA1	Glucose oxidase	7.54	0.03	0	3.2
1qanA1	Rrna Methyltransferase Ermc'	RRNA_A_DIMETH	1i8tA1	UDP-galactopyranose mutase	7.54	0.04	0	4.1
2cut_1	Serine esterase	LIG_DEP	1jfrA1	Exfoliatus Lipase	7.43	0.17	0	5.3
1bp1_2	Bactericidal Permeability-increasing protein	LIG__PC	1fuoA10	Fumarase C	7.42	0.01	0	0.1
1hcy_4	Hexameric haemocyanin	LIG_NAG	2kinA2	Kinesin	7.42	0.01	0	2.2
1cpq_1	Cytochrome C	LIG_HEM	1wpoB1	Human Cytomegalovirus Protease	7.41	0.01	0	1.3
1inp_1	Inositol polyphosphate 1-phosphatase	IMP_2	1bgxT6	TAQ polymerase	7.38	0	0	0
1ksaA1	Bacteriochlorophyll A Protein	LIG_BCL	1xvaA1	Glycine N-Methyltransferase	7.27	0.02	0	1.3
1b63A1	MutL DNA mismatch repair protein	LIG_ANP	1wpoB1	Human Cytomegalovirus Protease	7.22	0.03	0	0.6
1e7uA1	Phosphoinositide 3-Kinase Inhibition	PI3_4_KINASE_1	1qi9B1	Vanadium Bromoperoxidase Soluble Quinoprotein Glucose	7.15	0.01	0	0.6
1a12A1	Regulator Of Chromosome Condensation (Rcc1)	RCC1_2	1cruB1	Dehydrogenase	7.06	0.08	0	0.4

In some cases we found a structural similarity between a protein with unknown function and two patches annotated with the same function, giving strength to the hypothesis of function-related similarity. The conserved hypothetical protein (Tm0667) from *Thermotoga maritima *(PDB code 1j6o) shows a structural similarity with surface patches of *E. coli *nucleotidyltransferase (1gupA2) and *Desulfovibrio gigas *rubredoxin:oxygen oxidoreductase (1e5dA4), both annotated with the iron binding ability. The *E. coli *lysozyme inhibitor (1gpq), whose function is still uncharacterized, may bind ATP given the similarity to the *Rattus norvegicus *6-Phosphofructo-2-Kinase/ Fructose-2,6-Bisphosphatase major patch (1bif_1) and the mouse Aaa ATPase P97 (second patch (1e32A2)).

For each described match we propose that the detected structural similarity reveals a function-related similarity. For each match we checked whether the similarity could have been detected by means of sequence similarity, as checked using BLAST and PsiBLAST, or structural comparison, as checked by means of SSM and PINTS. Our approach, that is based on comparison of local functional surface residues, independently on their sequence order, may overcome the limitations of current methods possibly due to our incomplete knowledge of the sequence/structure/function relationship or to convergent evolution. Even using PINTS, which is a tool similar in philosophy to our approach, the findings are different, suggesting that different tools may be complementary in the difficult task of protein functional annotation; on the other hand, this may also highlight the difficulty in evaluating the significance of local similarities that in many cases are restricted to a very small number of residues.

## Conclusion

The expected burst in the number of protein structures that are not associated to a biological function, stimulated by the structure genomics programs, has emphasized the need for tools to reveal structural regularities even in proteins that do not share sequence or fold similarity [1,45]. Protein structures selected in structural genomics projects usually share very little sequence similarity with the dataset of already characterized proteins [46]. Sequence analysis tools are therefore unsuitable for inferring their functions. Moreover, cases are known where active site residues are not conserved in proteins sharing a common structural fold; therefore, "traditional" structure comparison tools are also not always able to help in function-related annotation.

Using a fully automated procedure, we obtained a reliable library of protein annotated functional sites. A fast structural comparison algorithm allows the rapid scanning of one or more protein structures with the library looking for local structural similarities. This method is designed to help in functional annotation in *difficult *cases. Our annotated surface patches determination and comparison method offers a new and powerful resource for detecting related function among unrelated proteins, for proteins solved in structural genomics projects or for identifying new function-related sites on the surface of already characterized proteins. We have been able to provide one or more functional clues to a large set of novel proteins, and, where functional evidences are already known, our findings confirm them. Moreover, just as proteins with different sequence and fold can share a similar functional site, proteins with similar sequence and/or fold can have small local differences leading to a completely different function [1,21]. Our method, which is focused on a detailed analysis of functional sites, is able to successfully predict protein functions in these difficult cases. Therefore, it can be used in analyzing the complex evolutionary relationships among protein sequence, structure and function [47-49]. The complete list of the functional predictions that we obtained is accessible at URL ; the structurally similar residues are shown for each match, and the structural superposition can be viewed through the browser plug-in Chime or RasMol. A novel publicly available web server, PdbFun [50], has been developed to allow the on-line structural comparison of user-defined subsets of residues of protein chains, and pre-defined subsets, like the SURFACE library of annotated functional sites, will be provided.

## Methods

### Functional site library extraction and annotation

The SURFACE database [32] stores a library of 1521 annotated function-related surface regions obtained using the following procedure (described in Figure 1): first, the SURFNET algorithm [51] is applied to a non-redundant, representative list of around 2000 protein chains from the PDB database [52] (downloadable at ) in order to find all the surface clefts with a volume higher than an arbitrary threshold (200 Å^3^); then for each cleft, a surface *patch *is identified as a collection of solvent-exposed residues using the MASK algorithm (that is part of the SURFNET package); finally, we infer the function of such surface patches using two kinds of annotations: *ability to bind *(associated to surface patch residues that are contacting a bound ligand), and match with PROSITE or ELM [33,34] functional motifs. The *ability to bind *annotation is carried out selecting those residues within 3.5 Å distance from any of the atoms of a ligand found in the crystal structure. Whenever a single patch contains more than 75% of the ligand-contacting residues (62% of the cases), we assign the ligand binding ability to this surface cleft. Considering only large organic molecules and metal ions, the ratio of the ligands that can be unequivocally associated to a single patch raises to 78%. PROSITE annotations are achieved scanning the sequences of monomers in our dataset using the ScanProsite algorithm [53], finding 928 matches. 12 matches were found with the ELM [34] experimentally verified instances. We did not consider those patterns marked by PROSITE as unspecific. Moreover, we annotated only those residues that correspond to non-X positions in the regular expression and that are exposed to the solvent according to the NACCESS procedure [54,55]. Once the dataset chains have been annotated, we map the annotated residues on the structure and in the surface patches. Whenever a single patch contains more than 75% of the pattern exposed residues, we assign the function encoded by this pattern to the patch (43% of the cases).

### Structural comparison

A sequence/fold-independent algorithm was used for local surface comparison [32]. The algorithm starts from a *seed *match (a pair of residues in the query that can be found in the target, at the same distance and with similar physical and chemical characteristics). The structural superposition, obtained by the quaternions method [56] and assessed at each step by residue similarity and root mean square deviation (r.m.s.d.) of the matching residues, is extended adding neighboring residues to the *seed *match until r.m.s.d and residue similarity are under user-defined thresholds (we used a similarity at least equal to 0.3 for each added pair of residues, and an average similarity at least equal to 1.2, using the Dayhoff substitution matrix [57] and 0.8Å as maximum r.m.s.d.). We consider only structural matches that include at least a fixed fraction (50%) of functional annotated residues, to increase the likelihood that the structural match is a function-related match as well. The algorithm is very fast and explores all the combinations of similar/identical residues in a sequence-independent way. The score of the match is the number of residues that can be superposed within the defined similarity thresholds. The significance of the score is evaluated by calculating the Z-score over the score distribution of the query patch comparison with the whole dataset: for each match, the Z-score is computed as the difference between the score of the match and the average score of all the matches for the query patch, divided by the standard deviation.

In order to obtain an estimate of the number of *true positive *matches, defining a *true positive *match as a structural similarity that implies also a functional similarity, we checked if the two matching proteins share also: *(i) *a common Gene Ontology (GO) term; *(ii) *a common SwissProt keyword; *(iii) *the same Enzyme Classification (EC) number; *(iv) *the same functional annotation (i.e. the binding of the same ligand or a match with the same PROSITE or ELM pattern). Gene Ontology terms search is limited to molecular function or biological process annotations linked to PDB structures from the GOA project [35]. SwissProt [58] keywords were extracted from the SwissProt entries corresponding to the DBREF field in the PDB [52] files header. If this was not available, we extracted the sequence from the order of residues in the structure, then we looked for a close homolog (sequence similarity higher than 95% using BLAST) in the SwissProt database. Some keywords were excluded because not referring to protein functions (i.e. *Structural protein*, *Polymorphism*, *Alternative promoter usage*, etc.). Furthermore, we checked whether the two matching proteins share more than 40% of sequence similarity or the same fold using the SCOP structural classification [39] at the superfamily level. Our database is composed of patches extracted from a non-redundant list of structures, therefore these cases are infrequent.

## Authors' contributions

FF carried out the patches definition, extraction and annotation, and the structural genomics protein functional prediction strategy, and drafted the manuscript. GA is the author of the structural comparison algorithm and participated in the design of the project. AZ participated in the procedure for the validation of structural matches, and in the creation of a relational structure to store and spread the project results. MHC participated in the project design and coordination and helped to draft the manuscript. All authors read and approved the final manuscript.

**Table 3:** Function prediction for uncharacterized proteins. Functional annotated sites have been used to infer the function(s) of a large set of uncharacterized proteins, using similarity threshold values that have been successfully tested on a training dataset. Columns: *(i) *PDB code and chain name of structural genomics proteins; *(ii) *PDB code, chain name and surface patch serial number of the functional annotated patch; *(iii) *Functional annotation of the matching patch; *(iv) *Z-score of the match; *(v) *Number of aligned residues; *(vi) *Blast2 bitscore; *(vii) *Sequence similarity evaluated by means of the Needleman-Wunsch global alignment (using the EMBOSS package 59 application *needle*). *(viii) *SSM Q score; *(ix) *SSM P score; (x) SSM Z score.

Str.gen	SURFACE patch	Annotation	Z-score	Score	BLAST2	Seq Sim	SSM Q	SSM P	SSM Z
1rtyC0	1qlaC1	LIG_HEM	14	7	0	0.5	0.06	0	1.5
1mw5A0	1bgyC2	LIG_HEM	13	9	0	0.8	0.04	0	1.8
1vhqA0	1ct9A1	LIG_AMP	13	8	13.9	1.2	0.02	0	1.8
1vhsB0	1cjwA1	LIG_COT	13	9	13.1	0.6	0.45	3.2	5.6
1vhsA0	1qsmD1	LIG_ACO	12	9	11.9	35.3	0.41	2.3	4.7
1j2rC0	19hcA1	LIG_HEM	12	8	12.7	0.4	0.01	0	1.5
1oz9A0	1fy7A1	LIG_COA	11	7	14.6	1.5	0.04	0	0.4
1vimA0	1dqrA1	LIG_6PG	10	7	15.4	1.1	0.07	0	3.1
1vj1A0	1tsdA1	LIG_UMP	10	6	13.9	3.2	0.01	0	0.2
1rtyA0	1fps_1	POLYPRENYL_SYNTHET_2	10	7	16.2	15	0.05	0	3.6
1vhnA0	2dorA1	DHODEHASE_2	10	8	13.5	0.7	0.23	0	5.7
1vhkA0	1grj_1	GREAB_1	10	7	0	2.5	0.02	0	2.2
1k7kA0	1qd1B1	LIG_FON	10	6	12.7	1	0.03	0	0.5
1vhkC0	1qd1B1	LIG_FON	10	6	13.5	2.1	0.04	0	2.5
1vhcA0	1bmtA2	LIG_COB	10	8	15	3.7	0.06	0	2
1uf9A0	1esmA1	LIG_COA	10	8	13.9	0.4	0.11	0	4.2
1h2hA0	1ezfA1	SQUALEN_PHYTOEN_SYN_1	10	7	13.1	1.3	0.02	0	0.4
1j5pA0	1ezfA1	SQUALEN_PHYTOEN_SYN_1	10	7	13.1	1.5	0.02	0	1
1rcuB0	2tpsB1	LIG_TPS	10	7	13.9	4.2	0.08	0	1.8
1vhcA0	2tpsB1	LIG_TPS	10	7	16.2	7.9	0.32	0.1	4.2
1jriC0	1atiA1	AA_TRNA_LIGASE_II_1	10	6	14.2	6.6	0.02	0	2.2
1j9jA0	1ft1A6	PPTA	10	7	14.2	1.9	0.02	0	0.6
1j9kB0	1ft1A6	PPTA	10	7	14.2	1.9	0.01	0	0.7
1i36A0	1eluA5	LIG_PDA	9	6	13.9	0.5	0.04	0	1.2
1j6pA0	1bxoA1	ASP_PROTEASE	9	6	13.9	2.7	0.02	0	0.3
1p5fA0	1eyrA1	LIG_CDP	9	6	21.6	33.2	0.06	0	1.9
1kytA0	1drmA1	LIG_HEM	9	6	12.3	0.9	0.02	0	1.6
1l6rB0	1drmA1	LIG_HEM	9	6	0	0.9	0.02	0	0.8
1j6rA0	1pprM1	LIG_DGD	9	6	0	3.7	0.01	0	1.4
1p99A0	1dik_1	LIG_SO4	9	6	14.2	1.7	0.07	0	0.7
1j2rD0	1dbtA1	OMPDECASE	9	6	15	2.5	0.07	0	1.6
1ni9A0	1pkp_1	RIBOSOMAL_S5	9	6	15.4	18.8	0.03	0	2.6
1lxnA0	1eg7A4	FTHFS_1	9	6	13.5	3.4	0.02	0	2.1
1rtyA0	1cpcB2	LIG_CYC	8	6	0	3.3	0.06	0	0.9
1vhnA0	1rblA1	LIG_CAP	8	6	14.2	1.5	0.09	0	2.9
1rtyA0	2cmd_1	MDH	8	6	13.9	19.8	0.02	0	0.9
1vj1A0	1hdoA1	LIG_NAP	8	6	14.6	3.2	0.07	0	3.3
1nc5A0	1aorA1	LIG_PTE	8	6	14.6	0.5	0.01	0	0.5
1rtwA0	1ft1A2	PPTA	8	6	13.1	11.7	0.03	0	1.7
1pg6A0	1qs0A1	LIG_TDP	8	6	13.5	0.2	0.02	0	0.9
1vizA0	1ho4B1	LIG_PXP	8	6	13.9	0.2	0.02	0	4.4
1l5xA0	1knyA1	LIG_APC	8	5	0	9.7	0.03	0	1.5
1vh6B0	1b72B1	HOMEOBOX_1	8	5	0	21.6	0.06	0.4	2.9
1mwqB0	19hcA1	CYTOCHROME_C	8	6	0	3.5	0.02	0	0.8
1s0uA0	1tplA1	BETA_ELIM_LYASE	8	6	15	0.6	0.02	0	2.6
1ixlA0	1ksaA1	LIG_BCL	8	6	0	2.2	0.04	0	1.9
1ufaA0	1nstA1	LIG_A3P	8	6	15.8	2.3	0.02	0	0.7
1rvkA0	2mnr_1	LIG__MN	8	6	14.2	39.5	0.05	9.3	9.8
1rvkA0	2mnr_1	MR_MLE_2	8	6	14.2	39.5	0.05	9.3	9.8
1vh6A0	1rdzA2	LIG_AMP	8	6	13.1	1.7	0.02	0	1
1ns5A0	1qjbB4	LIG_SEP	8	5	0	0.8	0.02	0	1.6
1rtyA0	1bcfA1	BACTERIOFERRITIN	8	6	16.2	7.3	0.14	0	0.9
1vi3A0	1a44_2	PBP	8	6	38.9	31.7	0.24	1	4.7
1j74A0	1dat_1	FERRITIN_1	8	6	15.8	5	0.00	0	0
1j7dA0	1dat_1	FERRITIN_1	8	6	15.8	0.7	0.00	0	0
1pc6A0	1qq8A1	HEME_OXYGENASE	8	6	0	0.9	0.03	0	1
1htwA0	1a4sA1	ALDEHYDE_DEHYDR_GLU	7	6	15	1.7	0.04	0	2
1vhmA0	1f5mB1	UPF0067	7	6	120	52.7	0.64	10	9.3
1vhmB0	1f5mB1	UPF0067	7	6	121	53.3	0.63	11.6	10.1
1rvkA0	2mnr_4	MR_MLE_1	7	6	14.2	39.5	0.05	9.3	9.8
1j6oA0	1e5dA4	LIG_FEO	7	6	14.2	0.3	0.03	0	0.1
1vhmA0	9icwA8	DNA_POLYMERASE_X	7	6	0	0.8	0.03	0	1.5
1qyiA0	2scpA1	EF_HAND	7	6	0	5.7	0.02	0	1.1
1nkvA0	1dhs_2	LIG_NAD	7	6	0	2	0.04	0	0.3
1nigA0	1c8zA1	TUB_2	7	6	0	0.7	0.01	0	4
1gpqB0	1bif_1	ATP_GTP_A	7	6	0	2.5	0.03	0	1
1p9vA0	1cjcA1	LIG_FAD	7	6	14.2	3.5	0.01	0	0.6
1vhmA0	1cjcA1	LIG_FAD	7	6	14.6	0.5	0.02	0	1
1vhmB0	1cjcA1	LIG_FAD	7	6	14.6	0.7	0.01	0	0.2
1lqlA0	1i78A5	OMPTIN_2	7	7	0	2.2	0.03	0	0
